# Emergency Department Pediatric Mental Health Care Bundle and Family Quality of Life

**DOI:** 10.1001/jamanetworkopen.2025.48860

**Published:** 2025-12-09

**Authors:** Amanda S. Newton, Jianling Xie, Jennifer Thull-Freedman, Teresa Lightbody, Jennifer Woods, Antonia Stang, Kathleen Winston, Jacinda Larson, Bruce Wright, Michael Stubbs, Matthew Morrissette, Stephen B. Freedman

**Affiliations:** 1 Department of Pediatrics, Faculty of Medicine and Dentistry, University of Alberta, Edmonton, Alberta, Canada; 2 Departments of Pediatrics, Cumming School of Medicine, University of Calgary, Calgary, Alberta, Canada; 3 Departments of Pediatrics and Emergency Medicine, Cumming School of Medicine, University of Calgary, Calgary, Alberta, Canada; 4 Children, Youth, and Families—Addictions and Mental Health, Alberta Health Services; 5 University of Alberta Hospital & Stollery Children’s Hospital Emergency Departments, Edmonton, Alberta, Canada; 6 Alberta Children’s Hospital Research Institute, University of Calgary, Calgary, Alberta, Canada; 7 Department of Psychiatry, Cumming School of Medicine, University of Calgary, Calgary, Alberta, Canada; 8 Department of Psychiatry, Faculty of Medicine and Dentistry, University of Alberta, Edmonton, Alberta, Canada

## Abstract

This secondary analysis examines the findings of a nonrandomized clinical trial assessing family quality of life after an emergency department visit.

## Introduction

In a nonblinded, nonrandomized clinical trial, an emergency department (ED) mental health care bundle that deployed resources to assess patient needs and facilitated follow-up care was associated with higher child well-being 6 months after a visit but not 30 days after.^1^ Here we present findings from a planned analysis of a secondary trial outcome, family quality of life (FQOL), chosen because it improves when family needs are met.^2^ We hypothesized that the care bundle would be associated with higher change in FQOL 1 month after the ED visit.

## Methods

Following site ethics approvals, 1412 children (eFigure in Supplement 1) were enrolled from 2 pediatric EDs in Alberta, Canada over 2 periods: standard care (preimplementation: January 29, 2020, to January 31, 2021) and bundle delivery (implementation: July 1, 2021, to June 30, 2022) (protocol, Supplement 2). All care was delivered by health care professionals. Enrolled children were younger than 18 years with non–life-threatening mental and/or behavioral health concerns that did not require medical treatment or stabilization or have communication barriers.

FQOL was reported by caregivers using the Beach Center FQOL scale, a valid and reliable 25-item measure with 5 subdomains: parenting, emotional well-being, family interaction, physical or material well-being, and disability-related support.^3^ We conducted segmented regression analyses to assess the change in FQOL trends before and after bundle implementation. All analyses were performed using individual-level data without prior aggregation. For each model (overall FQOL and each subdomain), the dependent variable was the change in FQOL score from baseline to 1-month follow-up. The models included 3 time-related variables: time in months since study start (to estimate the preintervention trend), a postimplementation indicator (to estimate the level change), and time in months since bundle implementation (to estimate the change in slope relative to the preintervention period). The latter term represented an interaction between time and the postimplementation indicator, making this specification mathematically equivalent to explicitly including the interaction term. Models were adjusted for age, gender, diagnostic category, triage acuity, admission status, baseline FQOL score, and study site (Supplement 1). Patients were analyzed according to their enrolled study period, regardless of the care received or adherence to the study protocol. Statistical significance was defined as a *P* < .05; all analyses were 2-tailed. Reporting follows the TREND reporting guideline.

## Results

Enrolled children were predominantly girls (747 [52.9%]) and White (1012 [71.7%]) with a median (IQR) age of 13.0 (11.0-15.0) years. Caregivers provided FQOL data for 1005 of 1412 children (71.2%) (407 missing [28.8%]); children with missing data had unspecified gender identities and ethnoracial backgrounds.

We found no significant change in the mean overall FQOL change following bundle implementation (level change: 3.34 [95% CI, −0.12 to 6.81] points), and no difference in the monthly trend (slope change: −0.15 [95% CI, −0.52 to 0.21] points per month) (Table 1). Findings were similar across most FQOL subdomains, except disability-related support, which increased after implementation (1.21 [95% CI, 0.33 to 2.09] points).

**Table 1.** Multivariable Segmented Regression Results for Overall FQOL Scores^a^

Outcome	Adjusted regression coefficient (95% CI)	*P* value
Time variables		
Preimplementation slope (monthly trend), points/mo	−0.15 (−0.39 to 0.10)	.25
Change in slope (post vs pre), points/mo	−0.15 (−0.52 to 0.21)	.41
Change in level at implementation, points	3.34 (−0.12 to 6.81)	.06
Covariate		
Baseline score	−0.29 (−0.33 to −0.25)	<.001
Age, per year older	−0.004 (−0.24 to 0.24)	.99
Gender		
Girl	[Reference]	NA
Boy	0.21 (−1.15 to 1.57)	.76
Nonbinary	−1.67 (−4.54 to 1.20)	.25
Not specified	−2.65 (−8.48 to 3.17)	.37
Transgender	−0.12 (−3.80 to 3.56)	.95
Ethnic and racial background		
Black or Latin American^b^	4.10 (0.96 to 7.24)	.01
First Nations, Inuit, or Métis	0.34 (−2.05 to 2.72)	.78
Multiple backgrounds	0.95 (−1.36 to 3.26)	.42
Not specified	0.84 (−5.27 to 6.94)	.79
South, Southcentral, Southeast, or West Asian	−0.16 (−2.74 to 2.42)	.90
White	[Reference]	NA
Diagnosis^c^		
Behavioral or emotional disorder	−2.48 (−4.99 to 0.04)	.05
Mood disorder	−1.10 (−2.76 to 0.56)	.20
Neurotic, stress-related, or somatoform disorder	−0.68 (−2.31 to 0.95)	.41
Other assigned diagnosis^b^^,^^d^	−1.44 (−3.19 to 0.32)	.11
Self-harm not requiring medical care	−0.58 (−3.81 to 2.66)	.73
Suicidal ideation	−1.01 (−2.56 to 0.55)	.20
Visit acuity, per 1 CTAS category lower	−0.33 (−1.28 to 0.62)	.50
Admitted at the index ED visit	1.19 (−0.73 to 3.11)	.22
Study site		
Alberta Children’s Hospital	1.35 (−0.02 to 2.72)	.05
Stollery Children’s Hospital	[Reference]	NA

Abbreviations: CTAS, Canadian Triage and Acuity Scale; ED, emergency department; FQOL, family quality of life; NA, not available.

^a^ The dependent variable in each regression model was the change in score, calculated as the difference between the 1-month score and the baseline score at the index ED visit. For continuous covariates, the adjusted regression coefficient represents the mean change in FQOL difference per unit increase in the covariate. For categorical covariates, the adjusted regression coefficient represents the mean difference in FQOL change between the specified group and the reference category, after adjusting for other variables in the model.

^b^ Groups combined due to sample size.

^c^ The reference group is not having the particular diagnosis.

^d^ Includes other diagnosis related to mental health needs, unspecified mental disorder, mental or behavioral disorder due to substance use, disorder of personality or behavior, and disorder of psychological development.

Among covariates, higher overall FQOL at baseline was associated with lower FQOL change 1 month after the ED visit (Table 2). Children with Black or Latin American ethnoracial backgrounds experienced a higher overall FQOL change at 1 month (4.10 [95% CI, 0.96 to 7.24] points). Across subdomains, nonbinary gender identity and diagnosis of a behavioral or emotional disorder were associated with lower emotional well-being change, while Black or Latin American ethnoracial backgrounds were associated with higher change in emotional well-being and family interaction.

**Table 2.** Multivariable Segmented Regression Results for FQOL Subdomain Scores^a^

Outcomes	Family interaction		Parenting		Emotional well-being		Physical/material well-being		Disability-related support	
	Adjusted regression coefficient (95% CI)	*P* value	Adjusted regression coefficient (95% CI)	*P* value	Adjusted regression coefficient (95% CI)	*P* value	Adjusted regression coefficient (95% CI)	*P* value	Adjusted regression coefficient (95% CI)	*P* value
Time variables										
Preimplementation slope (monthly trend), points/mo	−0.04 (−0.13 to 0.04)	.29	−0.02 (−0.09 to 0.06)	.65	−0.0001 (−0.06 to 0.06)	>.99	−0.05 (−0.11 to 0.01)	.11	−0.03 (−0.09 to 0.03)	.33
Change in slope (post vs pre), points/mo	−0.004 (−0.12 to 0.12)	.94	−0.08 (−0.19 to 0.03)	.14	−0.02 (−0.11 to 0.07)	.67	0.03 (−0.05 to 0.12)	.44	−0.06 (−0.15 to 0.04)	.23
Change in level at implementation, points	0.53 (−0.61 to 1.66)	.36	1.00 (−0.01 to 2.02)	.05	0.02 (−0.82 to 0.86)	.96	0.14 (−0.66 to 0.95)	.73	1.21 (0.33 to 2.09)	.007
Covariate										
Baseline score	−0.36 (−0.41 to −0.32)	<.001	−0.38 (−0.43 to −0.33)	<.001	−0.34 (−0.39 to −0.30)	<.001	−0.30 (−0.35 to −0.26)	<.001	−0.44 (−0.49 to −0.39)	<.001
Age, per year older	−0.04 (−0.12 to 0.04)	.31	0.02 (−0.05 to 0.09)	.65	0.04 (−0.02 to 0.10)	.15	0.03 (−0.02 to 0.09)	.28	−0.02 (−0.08 to 0.04)	.61
Gender										
Girl	[Reference]	NA	[Reference]	NA	[Reference]	NA	[Reference]	NA	[Reference]	NA
Boy	0.15 (−0.30 to 0.59)	.52	0.25 (−0.14 to 0.65)	.21	0.15 (−0.18 to 0.48)	.38	−0.31 (−0.62 to 0.01)	.06	−0.03 (−0.37 to 0.32)	.88
Nonbinary	−0.67 (−1.62 to 0.28)	.17	0.33 (−0.52 to 1.17)	.45	−0.90 (−1.59 to −0.20)	.01	−0.60 (−1.27 to 0.08)	.08	−0.15 (−0.88 to 0.58)	.68
Not specified	−1.05 (−2.97 to 0.88)	.29	−0.93 (−2.64 to 0.79)	.29	−0.57 (−2.00 to 0.85)	.43	0.30 (−1.07 to 1.67)	.67	−0.41 (−1.89 to 1.07)	.59
Transgender	−0.05 (−1.26 to 1.17)	.94	0.13 (−0.95 to 1.22)	.81	0.55 (−0.35 to 1.45)	.23	−0.21 (−1.08 to 0.65)	.63	−0.31 (−1.25 to 0.63)	.52
Ethnic and racial background										
Black or Latin American^c^	1.24 (0.20 to 2.28)	.02	0.56 (−0.37 to 1.48)	.24	1.01 (0.25 to 1.78)	.01	0.51 (−0.23 to 1.25)	.17	0.55 (−0.25 to 1.35)	.18
First Nations, Inuit, or Métis	0.21 (−0.58 to 0.99)	.60	0.30 (−0.40 to 1.00)	.40	−0.06 (−0.64 to 0.51)	.83	−0.23 (−0.79 to 0.32)	.41	−0.17 (−0.77 to 0.44)	.59
Multiple backgrounds	−0.11 (−0.87 to 0.65)	.78	0.40 (−0.28 to 1.08)	.24	0.38 (−0.19 to 0.94)	.19	0.15 (−0.39 to 0.70)	.58	0.48 (−0.11 to 1.07)	.11
Not specified	0.20 (−1.82 to 2.21)	.85	0.69 (−1.11 to 2.49)	.45	0.61 (−0.88 to 2.10)	.42	−0.46 (−1.89 to 0.98)	.53	0.53 (−1.03 to 2.08)	.51
South, Southcentral, Southeast, or West Asian	0.21 (−0.65 to 1.06)	.63	0.61 (−0.14 to 1.37)	.11	−0.20 (−0.82 to 0.43)	.53	−0.26 (−0.86 to 0.34)	.40	−0.39 (−1.05 to 0.26)	.24
White	[Reference]	NA	[Reference]	NA	[Reference]	NA	[Reference]	NA	[Reference]	NA
Diagnosis^d^										
Behavioral or emotional disorder	−0.78 (−1.61 to 0.05)	.07	−0.48 (−1.22 to 0.26)	.21	−0.74 (−1.36 to −0.13)	.02	−0.37 (−0.96 to 0.22)	.22	−0.24 (−0.88 to 0.40)	.47
Mood disorder	−0.11 (−0.65 to 0.44)	.70	−0.11 (−0.60 to 0.38)	.66	−0.24 (−0.65 to 0.16)	.24	−0.25 (−0.64 to 0.13)	.20	−0.08 (−0.50 to 0.34)	.72
Neurotic, stress-related, or somatoform disorder	−0.03 (−0.57 to 0.51)	.91	−0.03 (−0.51 to 0.44)	.89	−0.11 (−0.51 to 0.28)	.58	−0.13 (−0.51 to 0.25)	.51	−0.06 (−0.47 to 0.36)	.79
Other assigned diagnosis^c^^,^^e^	−0.51 (−1.09 to 0.07)	.08	−0.20 (−0.72 to 0.31)	.44	−0.27 (−0.70 to 0.16)	.22	−0.22 (−0.64 to 0.19)	.29	−0.24 (−0.69 to 0.20)	.28
Self-harm not requiring medical care	−0.19 (−1.25 to 0.87)	.73	−0.20 (−1.14 to 0.75)	.68	0.33 (−0.46 to 1.11)	.41	−0.27 (−1.02 to 0.49)	.49	−0.27 (−1.10 to 0.55)	.52
Suicidal ideation	−0.17 (−0.68 to 0.35)	.53	−0.19 (−0.65 to 0.26)	.40	−0.11 (−0.49 to 0.27)	.56	−0.35 (−0.72 to 0.01)	.06	−0.04 (−0.43 to 0.36)	.85
Visit acuity, per 1 CTAS category lower	−0.01 (−0.33 to 0.30)	.94	−0.04 (−0.32 to 0.24)	.78	−0.07 (−0.30 to 0.16)	.54	−0.16 (−0.38 to 0.07)	.17	−0.04 (−0.29 to 0.20)	.72
Admitted at the index ED visit	−0.14 (−0.77 to 0.49)	.65	0.12 (−0.44 to 0.69)	.67	0.18 (−0.28 to 0.65)	.44	0.29 (−0.16 to 0.74)	.21	0.43 (−0.06 to 0.92)	.09
Study site										
Alberta Children’s Hospital	0.31 (−0.14 to 0.76)	.18	0.41 (0.0025 to 0.81)	.05	0.31 (−0.02 to 0.65)	.07	0.32 (0.0019 to 0.65)	.050	0.16 (−0.19 to 0.51)	.37
Stollery Children’s Hospital	[Reference]	NA	[Reference]	NA	[Reference]	NA	[Reference]	NA	[Reference]	NA

Abbreviations: CTAS, Canadian Triage and Acuity Scale; ED, emergency department; FQOL, family quality of life; NA, not available.

^a^ The dependent variable in each regression model was the change in FQOL score, calculated as the difference between the 1-month score and the baseline score at the index ED visit. For continuous covariates, the adjusted regression coefficient represents the mean change in FQOL difference per unit increase in the covariate. For categorical covariates, the adjusted regression coefficient represents the mean difference in FQOL change between the specified group and the reference category, after adjusting for other variables in the model.

^c^ Groups combined due to sample size.

^d^ The reference group is not having the particular diagnosis.

^e^ Includes: other diagnosis related to mental health needs, unspecified mental disorder, mental or behavioral disorder due to substance use, disorder of personality or behavior, and disorder of psychological development.

## Discussion

In this study, a care bundle designed to improve mental health care was not associated with improved caregiver satisfaction with family life 1 month after an ED visit. While the study took place during the COVID-19 pandemic, a time of worsened family functioning,^4,5^ that higher baseline FQOL was not even maintained after the ED visit suggests the bundle should be refined to include care components designed to immediately improve FQOL. Because mean baseline FQOL scores were comparable to mothers of children with mild-to-moderate disabilities,^6^ this revised care approach could also match supports to the intensity of need (eg, child’s acute symptoms, family functioning). Finally, that several patient characteristics were associated with higher and lower satisfaction suggests that tailoring care to the child or family context is important.

This study has several limitations to be addressed in future studies. Conducting trials in multicenter general emergency care settings will improve the scope of understanding of care bundle delivery and its effects. Additionally, reminding participants during data collection of the steps taken to ensure the confidentiality of personally sensitive information could improve data completeness.

## Notes

Supplement 1. **eFigure.** Study Flow Chart
eMethods.

Supplement 2.
Trial Protocol

Supplement 3.
Nonauthor Collaborators

Supplement 4.
Data Sharing Statement
