# Supplementary material for: Emergency Department Pediatric Mental Health Care Bundle and Family Quality of Life
Source: JAMA Netw Open. 2025 Dec 9;8(12):e2548860. doi: 10.1001/jamanetworkopen.2025.48860 (PMC12690423; doi:10.1001/jamanetworkopen.2025.48860)
Supplement: Supplement 1. — eFigure. Study Flow Chart eMethods. [file jamanetwopen-e2548860-s001.pdf]

# Supplemental Online Content

Newton AS, Xie J, Thull-Freedman J, et al. Emergency department pediatric mental health care bundle and family quality of life. *JAMA Netw Open*. 2025;8(12):e2548860. doi:10.1001/jamanetworkopen.2025.48860

**eFigure.** Study Flow Chart

**eMethods.**

This supplemental material has been provided by the authors to give readers additional information about their work.

© 2025 Newton AS et al. *JAMA Network Open*.

**eFigure.** Study Flow Chart

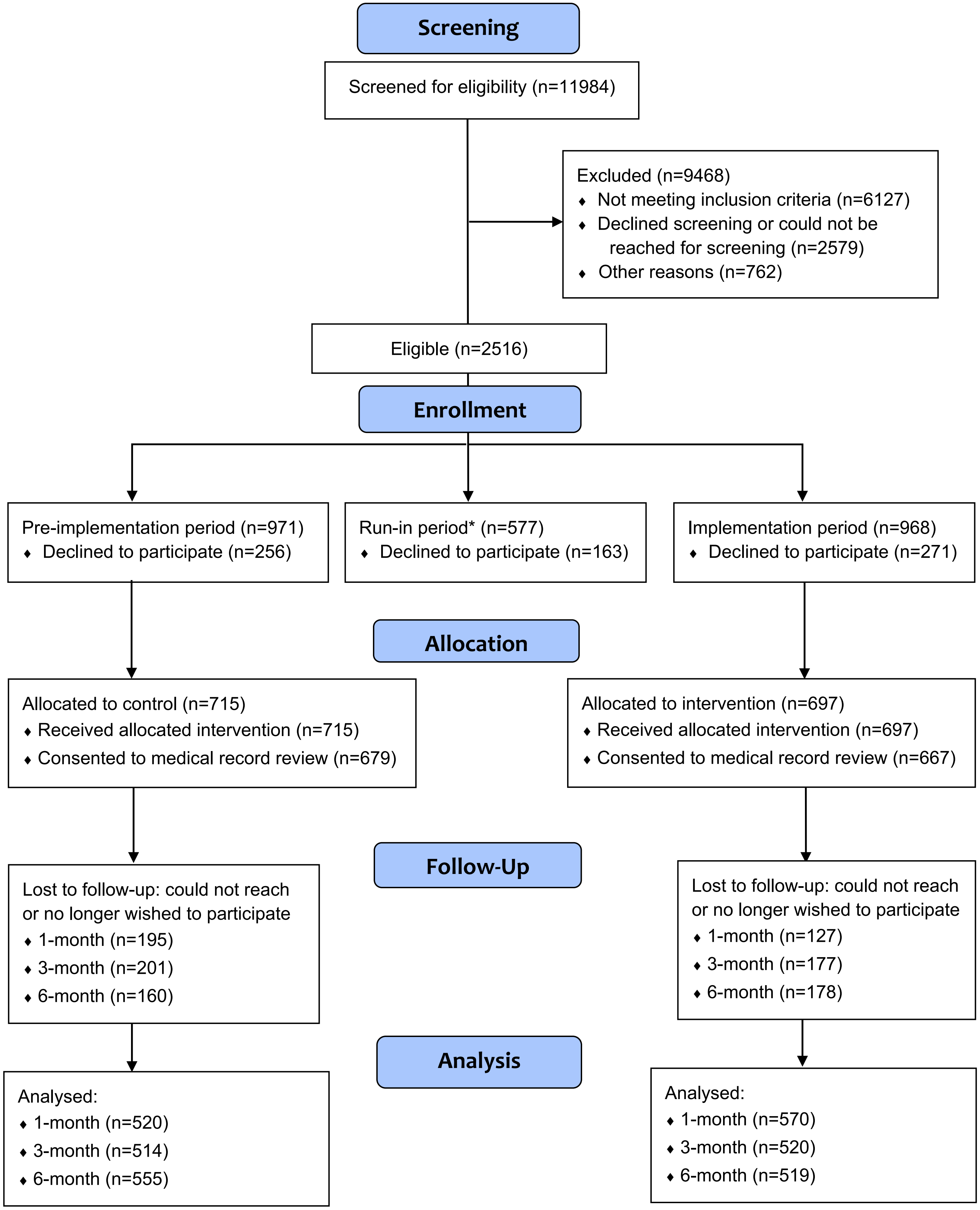


© 2025 Newton AS et al. *JAMA Network Open*.

* A 5-month period when care bundle components were introduced alongside training and resources to support implementation, and run charts were used with participant data to measure and improve adoption of bundle elements.

© 2025 Newton AS et al. *JAMA Network Open*.

**eMethods.**

**Statistical Modeling Approach**

We conducted segmented regression analyses at the individual level to evaluate changes in Family Quality of Life (FQoL) scores before and after implementation of the intervention bundle. The dependent variable was the change in FQoL score from baseline to 1-month follow-up. Separate models were fitted for the overall FQoL score and for each of the five subdomains of the FQoL scale. The models were fitted using ordinary least squares linear regression in R (function lm()). All analyses were conducted using individual-level data with no data aggregation prior to modeling.

**Time variables and coding**

- ***time in month*** is the number of months since the start of the study, coded as a continuous variable to capture the underlying temporal trend. For example, participants assessed in the first study month were coded as 1, those in the second month as 2, and so forth. This term represents the pre-intervention slope (monthly trend) in the change in FQoL in the model.
- ***post bundle implementation*** is an indicator variable (0 = pre-intervention; 1 = post-intervention) representing the level change at the time of bundle implementation.
- ***time in month after bundle implementation*** is a continuous variable coded as 0 for all pre-intervention observations and as the number of months since bundle implementation for post-intervention observations (e.g., 1 for the first month after implementation, 2 for the second, and so forth). This term estimates the change in slope (monthly trend) after the intervention relative to the pre-intervention trend.

**Covariates and coding**

- ***age***: Continuous (years).
- ***gender***: Categorical (male, female, nonbinary, trans-gender, not specified).
- ***ethnicity***: Categorical (Black or Latin American; First Nations, Inuit, or Métis; Multiple backgrounds; Not specified; South/Southeast/West Asian; White).
- **diagnosis:** Each coded as an independent binary indicator (1 = diagnosis present; 0 = not present) for suicidal ideation, F40–F48 (anxiety and stress-related disorders), F30–F39 (mood disorders), F90–F98 (neurodevelopmental disorders), self-harm, and other combined psychiatric diagnoses
- ***triage acuity***: Categorical (Canadian Triage and Acuity Scale levels 1–5, with 1 indicating highest acuity).
- ***admission status***: Binary (1 = admitted, 0 = not admitted).
- ***baseline FQoL score***: Continuous.
- ***Site***: Categorical (Alberta Children’s Hospital, Stollery Children’s Hospital).

**Interpretation of the time variables**

- ***time in month***: Estimates the pre-intervention slope, that is, the mean monthly change in FQoL score (points per month) before bundle implementation, adjusting for covariates.
- ***post bundle implementation***: Estimates the level change (points) in FQoL score at the time of bundle implementation, adjusting for all other covariates.

© 2025 Newton AS et al. *JAMA Network Open*.

- ***time in month after bundle implementation***: Estimates the change in slope (difference in monthly trend, points/month) following the intervention compared with the pre-intervention slope, adjusting for covariates.

© 2025 Newton AS et al. *JAMA Network Open*.
