# Supplementary material for: Emergency Department Pediatric Mental Health Care Bundle and Family Quality of Life
Source: JAMA Netw Open. 2025 Dec 9;8(12):e2548860. doi: 10.1001/jamanetworkopen.2025.48860 (PMC12690423; doi:10.1001/jamanetworkopen.2025.48860)
Supplement: Supplement 2. — Trial Protocol [file jamanetwopen-e2548860-s002.pdf]

UNIVERSITY OF CALGARY MEDICINE

UNIVERSITY OF ALBERTA
FACULTY OF MEDICINE & DENTISTRY

An Innovative Model of **P**ediatric **AC**ute **M**ental Health and **A**ddictio**N**s Care to Increase Value to Children, Youth, and the Healthcare System: The PACMAN Study

Protocol

Funded by:

Alberta Innovates – Partnership for Research and Innovation in the Health System (PRIHS)

Study Sponsors:

University of Alberta – Dr. Amanda Newton

University of Calgary – Dr. Stephen Freedman

Principal Investigators:

Dr. Amanda Newton

Dr. Stephen Freedman

UNIVERSITY OF CALGARY MEDICINE

UNIVERSITY OF ALBERTA FACULTY OF MEDICINE & DENTISTRY

UNIVERSITY OF CALGARY MEDICINE

UNIVERSITY OF ALBERTA FACULTY OF MEDICINE & DENTISTRY

Table of Contents

1. OVERVIEW ... 1

1.1 Need for improvement ... 1

1.2 Overview of our innovation ... 1

2. INTERVENTION AND PROPOSED CHANGES TO CLINICAL CARE ... 2

2.1 Improvements to triage in the ED ... 2

2.2 Improvements to ED care ... 4

2.3 Improvements in transitions between healthcare sectors ... 4

2.4 Phases of bundle implementation at the study sites ... 5

Pre-implementation phase ... 5

Implementation phase ... 5

Maintenance phase ... 6

3. RESEARCH DESIGN ... 6

3.1 Design ... 6

3.2 Participating centres ... 6

3.3 Patient allocation ... 6

3.4 Bundle inclusion criteria ... 6

3.5 Bundle exclusion criteria ... 7

3.6 Patient recruitment plan ... 7

3.7 Consent and assent ... 8

Capacity and mature minors ... 9

Waiver of Consent ... 9

3.8 Outcomes and measures ... 10

Patient-reported outcomes and experiences ... 10

Process/health system outcomes ... 11

Cost outcomes (Economics Sub-Project) ... 12

Balancing measures (Economics Sub-Project) ... 12

3.9 Data collection process ... 12

Administrative ... 12

Patient-reported ... 13

3.10 Sample size ... 13

3.11 Data analysis ... 13

Child/youth wellbeing and family functioning ... 13

Satisfaction ... 14

Receipt of follow-up mental healthcare ... 14

Process Outcomes ... 14

Health Economic Analyses (separate project) ... 14

4. COMMUNICATION PLAN ... 14

4.1 Year 1 ... 15

4.2 Year 2 ... 15

4.3 Year 3 ... 15

5. PATIENT ENGAGEMENT ... 15

6. CHANGE MANAGEMENT ... 16

6.1 Phase 1: Coherence (Year 1) ... 16

Change management plan ... 17

Communication plan ... 17

6.2 Phases 2 and 3: Cognitive participation and collective action (Year 2) ... 17

Among key individuals in EDs, new acute care clinics, and AHS mental healthcare clinics ... 17

Among key individuals working in mental health and addictions services offered in primary care, schools, community-based and non-profit organizations ... 18

6.3 Phase 4: Reflexive monitoring (Year 3) ... 18

7. ETHICAL CONSIDERATIONS ... 18

UNIVERSITY OF CALGARY MEDICINE

UNIVERSITY OF ALBERTA FACULTY OF MEDICINE & DENTISTRY

1. OVERVIEW

1.1 Need for improvement

For people in acute mental health or addictions crisis, Alberta's emergency departments (EDs) are vital navigational and safety net hubs for assessment, treatment and referral to specialized services. In the past 3 years alone, the annual number of ED visits in Alberta has increased by 16% for children and youth seeking mental health and addictions care (from 10,977 visits to 12,702 visits). The greatest numbers of visits occur in Alberta's two children's hospitals—in 2017, EDs in the Alberta Children's Hospital and the Stollery Children's Hospital saw 2,406 and 2,397 visits, respectively, for mental health and/or addictions concerns. This study focuses on improving care in these two EDs and their connections to community-based follow-up after the ED visit.

Child and youth mental health and addictions crises vary. Common reasons for seeking ED care are suicidal intentions, panic attacks, illicit drug use, and aggressive behaviours that risk hurting others. For children, youth and their families, these crises are stressful, can be overwhelming, and leave the family vulnerable. In response, acute care must be specialized and multidisciplinary. To be more than a temporary patch, this care must also integrate with broader services such as primary healthcare and community- and school-based services. Current clinical care and health system gaps in Alberta, however, prevent many children and youth from receiving specialized and integrated care.

1.2 Overview of our innovation

Our team has co-designed with patient partners an **acute mental healthcare bundle** that combines well-vetted solutions to improve healthcare quality, efficiency, and equality. In this study, we will implement and evaluate these solutions together for the first time, to achieve system-wide transformation and improved patient-level outcomes.

The acute mental healthcare bundle brings together multiple evidence-based assessment tools. We bundle tools that are used in isolation in other emergency care settings—Ask Suicide-Screening Questions and HEADS-ED—in consultation with their developers. Together, these tools ensure comprehensive, efficient, high-value ED-based assessment that is family-centred.

The bundle removes barriers to assessment and builds connections to care. The bundle shifts crisis assessment from an ED physician to a mental healthcare provider, introduces direct access to follow-up care, and strengthens sharing of information between care services.

The bundle introduces a shared decision-making framework for family engagement. For our bundle to be more than a temporary patch, we introduce Choice from the Choice and Partnership Approach (CAPA) to urgent care clinics. CAPA has been implemented in diverse mental health services, cultures, and healthcare systems. Improvements include increased family engagement in the healthcare system, reduced wait times to service, high satisfaction with care, and reduced ED use.

To determine the bundle's impact on children/youth, their families, and the healthcare system, the objectives of this study are to determine if the bundle:

1. **Improves child/youth wellbeing** (measured using the Stirling Children's/Warwick-Edinburgh Mental Wellbeing Scales)
2. **Improves child/youth and family satisfaction with care** (measured using the Service Satisfaction Scale)

3. **Improves family functioning** (measured using the Beach Center Family Quality of Life Scale)
4. **Improves healthcare delivery** (by measuring ED length of stay, access and time to follow-up care, ED revisits)
5. **Reduces and shifts costs**

2. INTERVENTION AND PROPOSED CHANGES TO CLINICAL CARE

The acute mental healthcare bundle builds on existing services and committed healthcare resources and investments. *Figure 1* on page 3 outlines how the bundle will be implemented.

2.1 Improvements to triage in the ED

In the bundle, triage nurses will use the Ask Suicide-Screening Questions. The ASQ tool is validated for pediatric ED patients and is used in many EDs across the US based on its ability to accurately screen for suicide risk among children and youth seeking care. Because healthcare providers differ in how they ask questions, and patient responses are influenced by how questions are posed, a standard approach to risk screening using a robust tool (i.e., the ASQ) identifies patients at risk more accurately. For triage nurses who feel ill-equipped about assess a child/youth's risk, the ASQ is an important tool. In hospitals that use the ASQ, staff report being very comfortable asking the questions. The tool requires minimal training. The goal of the screening is to identify children and youth who require further ED-based assessment to determine the actual level of risk.

The ASQ consists of four yes/no questions, takes 20 seconds to administer, and is available in 13 languages. A child/youth who answers, "Yes" to any question or refuses to answer one, is considered to be 'high risk'. Such children/youth have a threefold high risk for suicide. Children who reply "No" to all questions are considered to be 'low-risk'.

At Alberta Children's Hospital, children and youth who screen low-risk on the ASQ will be given a **booked appointment within 24-48 hours at an urgent care clinic** (a partnered mental healthcare clinic located nearby that permits real-time scheduling by ED staff). This approach will ensure families can discuss their needs and concerns—this currently does not occur for low-risk concerns. Families will meet with a nurse or social worker to review the details of their appointment (e.g., date, time, location, and name of clinic staff), and will leave the ED within 30 minutes of arrival.

At Stollery Children's Hospital, children and youth who screen low-risk on the ASQ will be able to go to the co-located mental health clinic (located in the hospital) during the clinic's hours. During off-hours, these children and youth will be given a **booked appointment within 24-48 hours at an urgent care clinic** (a partnered mental healthcare clinic located nearby that permits real-time scheduling by ED staff).

UNIVERSITY OF CALGARY MEDICINE

UNIVERSITY OF ALBERTA FACULTY OF MEDICINE & DENTISTRY

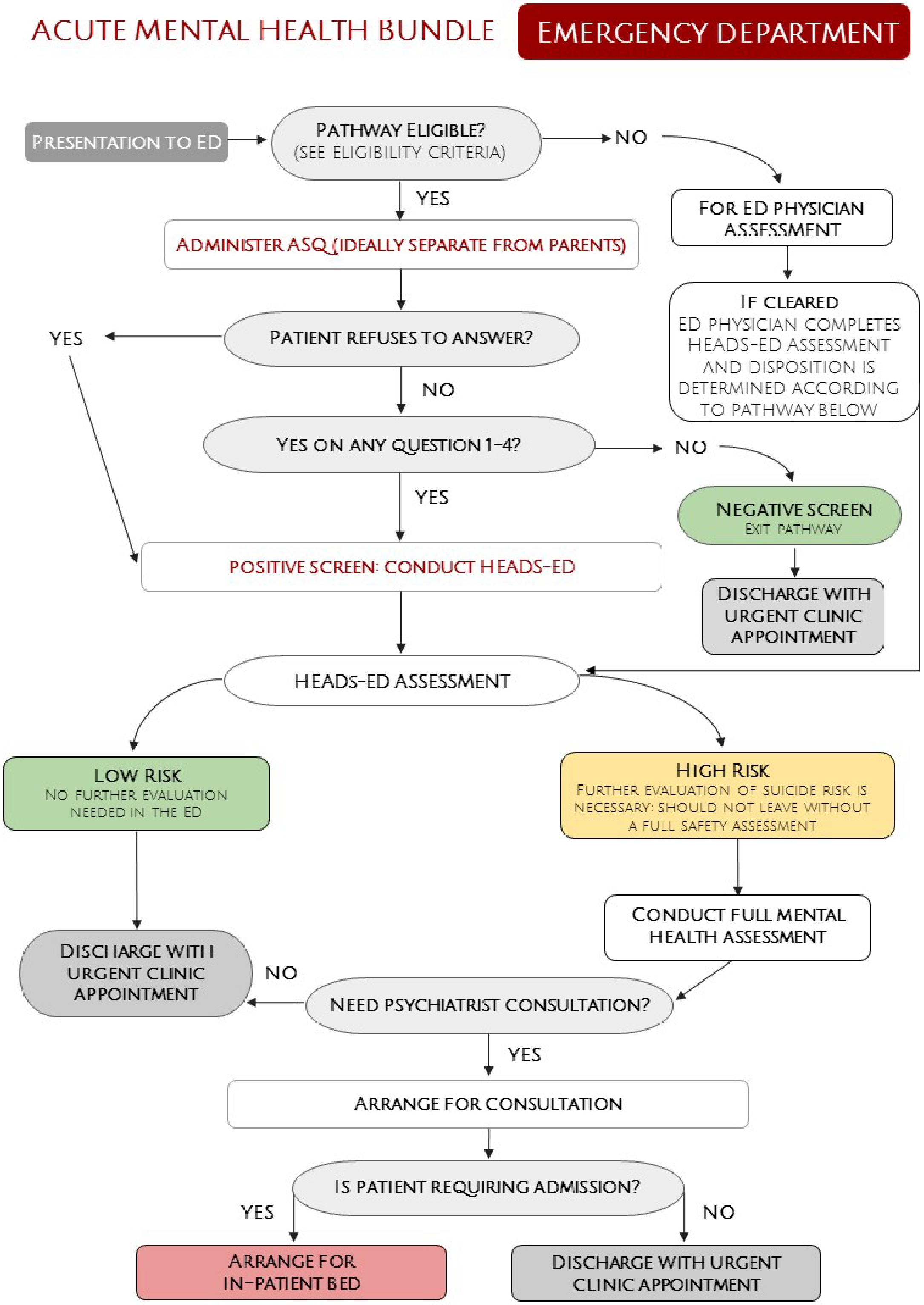

Figure 1. Care bundle implementation

2.2 Improvements to ED care

In the acute mental healthcare bundle, children and youth who screen high-risk on the ASQ **will not undergo medical clearance**. They will directly see a mental healthcare provider (e.g., psychiatric nurse, social worker) for a focused mental health assessment using the HEADS-ED tool.

The HEADS-ED mnemonic (Home, Education, Activities/Peers, Drug/Alcohol, Suicidality, Emotions and Behaviour, Discharge Reasons) is recommended for ED-based assessments. It improves communication, expedites decision-making, and guides the use of healthcare resources. Using the tool requires minimal training, and on average, should focus the mental health assessment so that it takes ~20 minutes to complete.

The HEADS-ED tool generates a total score and *action level* for the child/youth's concerns by summing the scores in each area in the mnemonic: 0 (*no/minimal functional impairment and no need for action*), 1 (*moderate functional impairment and needs action but not immediate*), or 2 (*severe functional impairment/needs immediate action*).

A total sum score <8 or 'suicidality' score of 0 or 1 will result in a 24-48 hour follow-up appointment in an urgent care clinic. ED physicians will provide medical evaluation if a concern emerges during the assessment or at the request of the family. This approach to assessment is expected to expedite mental health assessment, reduce ED physician costs, shorten ED length of stay, and provide families with follow-up after the ED visit.

2.3 Improvements in transitions between healthcare sectors

Currently, there is no standard access to follow-up care and recommendations are often non-specific. The care bundle will introduce Choice from CAPA to urgent care clinics. CAPA is a system transformation model with the goal of improving the flow of mental healthcare delivery through shared decision-making with families. Urgent care clinic resources (i.e., human resources and space) are readily available to support this transformation. In the bundle, Choice will deliver *the right mental healthcare, with the right people, at the right place, at the right time for families*. On the whole, CAPA has been shown to increase family engagement, reduce wait times, result in high satisfaction with services, and reduce ED use. CAPA is grounded in demand/capacity theory and Lean to smooth service flow, creating better patient and staff experiences. Full booking and streamlining of referrals will facilitate 24-48 hour follow-up care in urgent care clinics for children and youth discharged from the ED.

Using CAPA, the follow-up appointment in the urgent clinic will be a *Choice* appointment. It will focus on: a) understanding the main concern(s) and the child/youth/family's strengths, and b) developing joint goal setting and treatment planning. The appointment will conclude with a joint decision on what resources and services best match the family's context, needs, and goals. A *Partnership* appointment (treatment) can be booked as required. This prioritizing of *choice* promotes partnerships between a range of services for families—primarily healthcare, school, social and community-based services. Unique needs and preferences (e.g., newly immigrated, LGBT2Q, financial) are considered, improving the family experience. During this project, as needed, urgent care clinic staff will expand on existing processes for care coordination between services to support interagency connections and seamless transitions. As a result, families will be directly connected to and not required to self-advocate to receive the care they need.

2.4 Phases of bundle implementation at the study sites

Operational team leads will actively manage bundle implementation including how PDSA cycles will be used to refine and improve bundle components, change management activities (Section 6), and communication (Section 4).

Pre-implementation phase

Sites will begin with a 12-month period where there are no active, systematic, mental healthcare delivery innovations introduced. This phase will serve to provide the collection of baseline outcome measurements. During the last 2 months of this phase, a change management team at each site will follow Prosci®; a structured methodology with practical tools to support changes in healthcare practices. To help teams organize the phases of change during bundle implementation, we are using Normalization Process Theory. Each site will include a change management lead, change specialist, 6 project team members, and 4 site champions. ED and urgent care clinic managers, patient care managers, nurse educators, and senior leaders will also be involved. The change specialist will work with site champions on training plans and change management strategies, identifying how the care bundle will impact individuals. Strategies will be informed by identifying individual and organizational risks, barriers, and enablers, and supported by a change management communication plan. The change specialist will create change management process maps, champion roadmaps, coaching and training plans for frontline providers, and a resistance management plan. These tools will support implementation in the next 2 phases. The change specialist will also create an electronic database to track adoption issues and progress. Completed change management components will be integrated into the project plan before implementation and reviewed quarterly for responsiveness to individual/organizational needs.

Implementation phase

This phase will last 5 months at each site. Sites will introduce the care bundle, applying a quality improvement (QI) approach through iterative PDSA cycles. The purposes of PDSA cycles are to: (a) learn as quickly as possible whether changes lead to desired improvements in different settings; and (b) make adjustments accordingly, increasing our ability to confidently deliver and sustain impacts. Integrated data management will offer real-time outcome monitoring. Cycles will optimize system performance and ensure structure to bundle testing. With PDSA cycles, our team will be able to quickly determine if changes lead to desired improvements and make adjustments to optimize performance. Each cycle will test a change aimed at bundle improvement: planning the change based on change theory and previously collected data (Plan), enacting the change (Do), examining change impact (Study), and identifying learning to inform next steps (Act). We will carry out multiple cycles and analyze data from each cycle to detect system improvement. As cycle example, we could test the impact on ED length of stay by removing medical clearance by the ED physician. After 1 week of measuring length of stay and obtaining staff and family feedback, the bundle would be modified to incorporate learning and then tested on a larger group of physicians before permanent implementation. By assessing the impact of changes in real time starting with small patient samples, we will address challenges, reduce unintended consequences, and maximize staff and family engagement. This cyclical approach will mitigate risks in implementing untested changes. Successful change management within a system will be supported by: a) demonstrating need for change, b) engaging staff impacted by implementing changes, and c) creating evidence of success and sustainability. Findings from each cycle will be documented to support local learning and transferability of learning across sites.

During PDSA cycles, we will track change management activities. At the completion of each cycle, the site-specific change management strategy will be reviewed and refined. We have organized special teams at each site to oversee the cycles and change management plan:

- **Operational team leads** helped develop critical aspects of our implementation plan and will manage bundle implementation.
- **Quality improvement leads** will ensure PDSA cycles follow organizational quality management processes. These leads will also guide how findings from PDSA cycles will be used to refine and improve operationalized components of the bundle.
- **Change specialists** will consult with healthcare providers, unit/patient care managers and site champions to identify how practices/processes are influenced over time. Resistance or lack of engagement will be explored and addressed via change management process maps, champion roadmaps, coaching and training plans for frontline providers, and a resistance management plan.

Maintenance phase
In the final 12 months, we will stop testing and changing the bundle with PDSA cycles. We expect to achieve maximal system uptake and efficiency by the end of the implementation phase. During maintenance, we will monitor bundle performance with ED dashboards. Change specialists and site champions will use performance measures and site-specific change management strategies to ensure fidelity to the care bundle is high. Champions will share findings in real time with healthcare providers to further promote adherence to the bundle.

3. RESEARCH DESIGN

3.1 Design

To measure the impact of the acute mental healthcare bundle, we will use a quasi-experimental design allows us to study a complex intervention under 'real world' conditions so that operational leads have results that are relevant and usable. Data will be collected longitudinally before and after introducing the bundle to services; this approach will allow us to examine, using an interrupted time series approach, how the bundle influenced outcomes relative to the underlying secular trend and competing non-contemporaneous interventions.

3.2 Participating centres

This study involves 2 'bundle sites': Stollery Children's Hospital, Edmonton; Alberta Children's Hospital, Calgary.

3.3 Patient allocation

Patients will not be randomized in this study. Care received will be dictated by implementation phase at the two bundle sites (pre-implementation, implementation, maintenance).

3.4 Bundle inclusion criteria

Children and youth eligible to receive the care bundle will be identified by nurses at triage. Eligible children/youth must be aged <18 years and have a mental health complaint in one of the following categories:

a) Anxiety
b) Bizarre behaviour
c) Concern for patient's welfare

d) Deliberate self-harm
e) Depression/suicidal
f) Homicidal behaviour
g) Insomnia
h) Pediatric disruptive behaviour
i) Situational crisis
j) Violent behaviour

We will create a pathway for ED healthcare providers to refer to that will describe bundle eligibility criteria in detail. All bundle-eligible children/youth will be triaged, registered, have charts and contact sheets created, and receive care that follows the newly created bundle pathway (see Figure 1). Families opting to stay in the ED to be seen will have triage completed and will be seen as per regular triage priorities and will initially be seen by an ED physician with mental health consultation performed as indicated.

3.5 Bundle exclusion criteria

Children and youth not eligible to receive the care bundle will receive acute care following institutional policies. Approximately 20% of all mental health visits (based on site-specific data and the literature) will be by children/youth in an ineligible category:

a) Brought to the ED by police or peace officers – they bypass triage, go straight to a room, are often restrained, are being held involuntarily, and require urgent physician assessment
b) Held under Form 10
c) Features of schizophrenia, schizotypal and delusional disorders (psychosis)
d) Behavioural syndromes associated with physiologic disturbances (e.g., eating disorders)
e) Significant intentional self-harm (e.g., laceration, ingestion, hanging)
f) Previous participation in the study

Patients who do not meet the inclusion criteria and/or who are excluded based on at least 1 exclusion criterion will undergo traditional local care approaches that include physician assessment following triage in keeping with current standards of ED care. In addition, at any point in time, should the patient/family/healthcare team desire deviation from the acute mental healthcare bundle, the family can be switched into the traditional ED care track and based on acuity/urgency, an ED physician will provide a medical evaluation.

3.6 Patient recruitment plan

Study recruitment will not occur during the ED visit. Patient partners have told us they do not want to be approached regarding research during the ED visit – therefore will therefore conduct daily reviews of ED medical records at both sites to identify all children and youth eligible for the study. A standardized screening and eligibility confirmation form will be used to guide decision making around study eligibility within REDCap. ED medical records will be reviewed of all children/youth who have a mental health and/or addictions related discharge diagnoses. One of the following CEDIS chief complaints in a child/youth's medical record will indicate potential eligibility:

a) Anxiety
b) Bizarre behaviour

c) Concern for patient's welfare
d) Deliberate self-harm
e) Depression/suicidal
f) Homicidal behaviour
g) Insomnia
h) Pediatric disruptive behaviour
i) Situational crisis
j) Violent behaviour

Consistent with criteria used to determine bundle ineligibility, a child/youth with the following diagnoses in the medical record will not be contacted to participate in this study:

a) Brought to the ED by police, peace officers, or ambulance
b) Held under Form 10
c) Features of schizophrenia, schizotypal and delusional disorders (psychosis)
d) Behavioural syndromes associated with physiologic disturbances (e.g., eating disorders)
e) Significant intentional self-harm (e.g., laceration, ingestion, hanging)
f) Previous participation in the study

Multiple approaches will be employed to obtain permission for the research team to contact the potential study participant:

1) An agent of AHS will approach potentially eligible patients and families in the ED prior to discharge to provide a study information sheet, consent form, and obtain consent to contact.
2) For patients who are missed in the ED, we will use the contact information that is collected at ED registration to phone parents/caregivers of eligible children/youth and obtain consent to contact by an agent of AHS. If consent to contact is successfully obtained, appropriate contact information will be collected and documented in this phone call and passed on to the research team.

A research assistant will phone parents/caregivers 24-72 hours after the ED visit to complete recruitment for the study. A standardized script, approved by each institution's ethics board, will be used to obtain written consent. All patients/families who consent to participate in the study will contribute administrative and survey data. Participants can consent verbally to the collection of survey data during the recruitment phone call. Written consent will be required prior to the collection of administrative data from the medical record.

3.7 Consent and assent

Three methods will be utilized individually and/or in tandem to facilitate the collection of written informed consent and assent:

(1) **Written Consent:** Participants may sign the hard copy of consent/assent provided by an agent of AHS in the ED. This can be returned to the research team by taking a photo of the signature page and emailing to a secure address. Alternatively the document can be returned by standard mail or email (ie. scanned PDF) to the team.
(2) **Electronic Consent:** Participants may electronically provide written consent/assent to study participation when contacted by the study team by telephone for recruitment. If this method of consent is chosen by the participant, the research assistant will send a unique URL to a verified

email address or through a text message (utilized from within REDCap) that will link to the REDCap database and allow for the completion of electronic consent.

(3) **Verbal consent + Written/Electronic Consent:** Participants may provide verbal consent to participating in the survey portion of the study when contacted by the study team by telephone for recruitment. In addition, one of the above two options must be utilized to provide written consent prior to the collection of any health information from the medical record.

All study team contacts with the potential participant will be in accordance with what is outlined in the consent to contact form provided by the agent of AHS. Where applicable, research assistants will ensure copies of the verbal informed consent/assent documents are stored in a locked cabinet and a secure room at each respective study site. The research team will be the only individuals with access to this secure area. At the end of the study, all records will continue to be kept in a secure location for as long a period as dictated by the reviewing research ethics board (minimum of 5 years).

Capacity and mature minors

As indicated by the University of Calgary Conjoint Health Research Ethics Board, youths that are 14 to 17 years old may be considered mature minors if they have sufficient decision-making capacity. For the purposes of this study, whether the youth will be treated as a mature minor will be determined when the agent of AHS approaches to obtain consent to contact. If the family believes the youth to be the appropriate contact, the research team will treat the youth as a mature minor and the primary contact for the study. In the case of youths 14 years of age or older who attend the emergency department without the presence of a parent or legal guardian and provide their personal contact information when consenting to contact, the study will automatically treat them as mature minors.

When the research team contacts potential participants for recruitment, standard procedures for assessing capacity to consent will be employed. When determining capacity, research staff will assess the following:

- The intelligence of the minor;
- The minor's ability to appreciate the reasonably foreseeable consequences of his or her actions;
- The stage of the minor's physical, emotional, and mental development;
- The degree of responsibility the minor has assumed in his/her life; and
- The inherent risk level of the research protocol

Upon reviewing the study with the minor, the research staff will assess the following:

- Does the minor have the ability to recall study details?
- Does the minor have the ability to identify potential study risks and benefits?
- Does the minor have the ability to formulate and ask informed questions about the study?

Waiver of Consent

For study participants who enrolled in two time periods, pre-implementation (pre-bundle implementation period) and maintenance (post-bundle implementation period), we will use a waiver of consent to link healthcare visit data outside of the index ED visit. These data will be from Alberta Health and will be used for the economic analysis sub-project (page 15) to understand the impact of the bundle. This sub-project will be submitted as a separate project to the Ethics Board but is noted here for comprehensiveness.

3.8 Outcomes and measures

We will target 3 groups of outcome measures—patient-reported, process, and cost—to generate a comprehensive set of data relevant to diverse stakeholder groups.

Patient-reported outcomes and experiences

The main outcomes of interest for this study include:

- Satisfaction with acute mental health and addictions care
- Consideration of child/youth and parent/caregiver preference for type of follow-up care
- Child/youth wellbeing
- Family wellbeing

Satisfaction with acute mental healthcare services: Satisfaction will be measured using the Service Satisfaction Scale (SSS-10), an instrument for measuring global satisfaction with mental health services [Appendices P and Q]. It has several advantages over other measures: brevity, parallel youth and caregiver forms, availability at no cost, and development with data from a large sample of youth and caregivers with rigorous psychometric methodology. It has been routinely employed to evaluate outpatient services in Alberta and was selected by our patient stakeholders over other options provided during stakeholder meetings. The scale consists of 12 items (parent version) or 10 items (youth version) that are relevant to a range of mental health services. Items are scored on a 5-point response scale, with a total possible score of 60 (parent) or 50 (youth) indicating high satisfaction. We will administer the SSS-10 within 24–72 hours of the index ED visit.

Consideration of child/youth and parent/caregiver preference for type of follow-up care: CAPA aims to match child/youth family needs and preferences to recommended services. We will measure whether the service met preferences, using a single SSS-10 item about involvement in treatment decisions: "Did you have enough involvement in decisions about your/your child's treatment?" (Responses: Yes, definitely; Yes, somewhat; No, I wanted to be more involved). The SSS-10 will be administered within 24-72 hours of the index ED visit.

Child/youth wellbeing: Wellbeing will be measured with the Stirling Children's Well-Being Scale (SCWBS) for children aged <13 years and the Warwick-Edinburgh Mental Wellbeing Scale (WEMWBS) for youth aged 13.0-17.99 years. Both scales are required because they are designed for different age groups. The scales assess wellbeing over the previous 2 weeks, are validated across many cultures, and are endorsed for measuring and monitoring wellbeing. We will administer the SCWBS/WEMWBS within 24-72 hours (baseline) of the child/youth's index ED visit and again at 1 month (primary outcome time-point). The 3-month and 6-month time-points will support assessment of longer-term improvement. These scales measure the same construct in 2 different age groups, so data will be standardized and then combined across age groups to derive a single wellbeing measure.

The SCWBS is a short, robust measure that asks children 12 questions about their emotional and personal wellbeing. The scale has been used to assess change in wellbeing in several studies. The SCWBS covers 3 domains (optimism, cheerfulness and relaxation; satisfying interpersonal relationships; clear thinking and competence) to form a single dimensioned scale of wellbeing. A 5-point response scale is used and scores range from 12-60 with higher scores representing a greater level of wellbeing. The scale was developed and standardized across 18 schools with 1849 children. It has excellent internal consistency (Cronbach's α=0.85) and good test-retest reliability (r=0.75).

The WEMWBS scale consists of 14 questions with 5 response categories. Scores range from 14 to 70, higher scores representing a greater level of wellbeing. Focus group discussions with youth suggest that WEMWBS items are clear, user friendly and unambiguous. The scale has been adopted to measure wellbeing among children and youth. It is responsive to changes in different populations across a range of mental health interventions. The scale has excellent internal consistency (Cronbach's α =0.89) and test-retest reliability (r=0.83). It has no ceiling or floor effects and is unsusceptible to bias. It has been validated in a general sample at population and individual level in the UK, and Europe and among ethnic minorities. The WEMWBS is valid and reliable when used with youth receiving secondary care (services for youth with severe mental health conditions that require emergency care or those unresponsive to primary care interventions).

Family functioning: Parent/caregiver members of our planning team stressed the importance of understanding our care bundle's impact on family functioning, with the goal of better functioning after a crisis. We will use the Beach Center Family Quality of Life Scale (FQOL) to measure parent report of family functioning. The FQOL is a concise 25-item instrument with questions on parenting, emotional wellbeing, family interaction, physical and material wellbeing, and disability related support. The scale has satisfactory psychometric properties. It uses a 5-point rating scale, with a maximum score of 125 indicating highest quality of life. We will administer the FQOL within 24–72 hours of the initial ED (baseline) visit and again at 1 month.

Process/health system outcomes

In this project, we will access administrative healthcare data for participant ED visits. ED data will be reviewed for participants who consent/assent to medical record review. We will measure the following outcomes:

- Number of children/youth per month discharged ASQ at triage (low-risk screening outcome) to urgent care follow-up clinic
- Number of children/youth per month who receive psychiatry consultation in the ED after HEADS-ED assessment
- Number of children/youth per month discharged to urgent care clinic after HEADS-ED assessment
- Number of children/youth per month discharged with another recommendation after HEADS-ED assessment, and documentation of the recommendation
- Number of ED visits per month for child/youth mental healthcare that concluded in admission to each children's hospital or transfers to admit at another institution
- Length of ED stay (the interval between time of ED arrival and time of ED departure, either admission or discharge; in minutes) for visits per month to each children's hospital
- Proportion of discharged children/youth who received urgent care clinic follow-up within 48 hours
- Number of child/youth receiving follow-up mental healthcare within 7 days of the ED visit (to be reported by parents/caregivers at 1-month follow-up)
- Number of child/youth receiving follow-up mental healthcare that relates to their index ED visit, within 7 days and 1 month of the index visit
- Appropriate care in the appropriate setting. Research assistants will audit all ED/clinic records monthly at each site and document whether assessments children/youth received are congruent with discharge recommendations
- Type of follow-up care accessed. Parents/caregivers will report types of follow-up care within a month after an ED visit or urgent clinic follow-up appointment

Cost outcomes (Economics Sub-Project)

This sub-project will involve a focused, conservative approach to costing by only examining child and youth mental healthcare use, not examining all service use. Investigators from the Institute of Health Economics will prepare a separate protocol for this sub-project.

Balancing measures (Economics Sub-Project)

Investigators from the Institute of Health Economics will document the number of ED revisits for mental and substance use disorders, suicide attempts (ED visits for self-harm), and completed suicides within 1 month of the index ED visit. ED revisits and suicide attempts will be determined using data provided from Alberta Health. The results will be shared with the PACMAN team to identify any unintended consequences of our care bundle.

3.9 Data collection process

We will use REDCap, a secure-web based application, to collect non-routine, outcome data from families (e.g., satisfaction, wellbeing). No additional data will be collected in the ED in order to minimize the impact on families during a time of crisis. Following the ED visit (24-72 hours later), using demographic data provided at registration, participants will be contacted at which time they will be asked to participate in this initiative. Eligible participants and/or their families may choose to decline and this will not affect their care. Participants who consent/assent can complete measures immediately. They will be offered the option of completing the data collection surveys by phone or electronically via REDCap (through their computer or mobile phone).

Administrative

Daily medical record review by research assistants will include collecting data on the number of bundle-eligible children/youth, number of children/youth with urgent clinic appointment booked based on ASQ screen at ED triage, and HEADS-ED assessment outcomes. Administrative databases will be accessed to collect: length of ED stay, time of admission to ED, time of discharge from ED, the Canadian Triage and Acuity Scale (CTAS) and the number of children/youth who received urgent care clinic appointment within 48 hours. Also, data will be collected for any ED visits within 30 days of the participants' index ED visit including the re-presentation visit triage date and time, the chief complaint, admission status, CTAS score, and discharge diagnosis. The Calgary team will access Edmonton's site participants' ConnectCare for the data collection within 30 days of the index ED and also the CTAS score from the index ED visit.

The PI will provide the full name, age, and Alberta personal health number (PHN) of study participants enrolled in the pre-implementation bundle and post-bundle implementation periods with Alberta Health to enable linkage to administrative healthcare databases in the economics analysis project (e.g., NACRS, ACCS, DAD, Alberta Health, Coroners' Data).

To enable the analysis in the economics sub-project, the PI will provide investigators at the Institute of Health Economics with study participants':

1. Study screening ID (generated for study administrative purposes)

2. Date of enrolment (to confirm study phase)

3. Assigned cohort (pre-implementation or post-implementation; to confirm study phase)

4. Age and gender

5. Forward sortation data (participant disclosed 3 first digits of postal code) at time of enrolment

6. Well-being scores

Patient-reported
Data will be collected electronically using REDCap or by phone. Participants will provide an email/phone number for email/text mailing of surveys or administration over the phone, as selected by participants. The table below summarizes collection from youth and parents/caregivers.

Baseline (24-72 hours post ED visit)	1-Month Follow-up	3-Month Follow-up	6-Month Follow-up
- SCWBS/WEMWBS<br>- SSS-10<br>- FQOL	- SCWBS/WEMWBS<br>- FQOL	- SCWBS/WEMWBS	-SCWBS/WEMWBS

3.10 Sample size
Each series in our design includes at least 8 measurement time-points (intervals). In 2017, the Alberta Children's Hospital and Stollery Children's Hospital provided mental healthcare to 150–200 children/youth per month. Based on an anticipated consent rate of 75% and 1-month follow-up rate of 75% during each phase, we will have ≥50 patients/interval. We conducted preliminary calculations for the series with the fewest number of data points (8) and patients (50/month) using a 2-sample t-test comparing mean values in the pre-implementation phase to those in the maintenance phase. These calculations assume no serial autocorrelation and a sustained effect of the bundle, which is justified based on the PDSA cycles that will have occurred. With at least 800 patients in both the pre-implementation and maintenance phases, respectively, we will have 90% power to detect an absolute improvement as small as 1.9 units on the SCWBS/WEMWBS (primary outcome), assuming a common standard deviation of 10 using a 2-sided 5% level of significance. Thus, we are well powered to detect a difference of 3 units at each study site, the minimally clinically important difference defined for this outcome. We are below the effect observed in other studies using the WEMWBS. These preliminary calculations will be updated once baseline data become available.

3.11 Data analysis
Child/youth wellbeing and family functioning
Data will be summarized and presented as mean scores per month per site. Visual inspection of the multiple baseline time series plots will be used to refine the analytical approach, i.e., to examine the type of secular trends, to check for the presence of seasonal effects and any outliers, and to examine heterogeneity in baseline levels and secular trends across the 2 study sites. Our anticipated primary analytical approach will use site-specific linear segmented regression to analyze trends over time. The model for each series will include terms for the baseline level and pre-intervention trend, change in intercept and change in trend from the pre-implementation phase to each of the implementation and the maintenance phases. Durbin-Watson tests will be used to test for serial autocorrelation, and the autocorrelation coefficient will be accounted for in the analysis where necessary. A change in intercept and/or slope from the pre-intervention to the maintenance phase in the intervention sites will provide strong evidence of an effect due to the intervention. If the observed level and trends are sufficiently similar across the two sites, we will conduct a pooled mixed-effects regression analyses across the two sites to generate a combined estimate of the intervention effect. We will account for key child/youth

features as covariates in the model (age, self-reported gender, ethnicity, and acuity of concern at the initial ED visit).

Satisfaction

Satisfaction scores will be summarized and presented as mean scores per month per site.

Receipt of follow-up mental healthcare

Descriptive statistics (e.g. frequencies, proportions) will summarize the number of children/youth who received and did not receive follow-up care.

Process Outcomes

Descriptive statistics (e.g. frequencies, proportions) will summarize process outcomes per month, which will be analyzed as described above for the wellbeing and functioning outcomes. Length of ED stay will be expressed as mean time (with interquartile range) and analyzed using segmented regression as described for the primary outcome (wellbeing).

Health Economic Analyses (separate project)

Health economic analyses will combine data on costs of resource use with outcomes on child/youth wellbeing to determine if our care bundle is cost-effective and gives value for money for public payers. The analyses will use SCWBS/WEMWBS scores to measure the benefit of our care bundle. Incremental cost and wellbeing outcomes will be combined to calculate incremental cost-effectiveness ratios. The change in costs minus cost of implementing the bundle will be reported as a return on investment statistic. Uncertainty will be addressed with bootstrap analyses.

We considered a range of quality of life measures but found them unresponsive to changes in mental health in our population, either because mental health items were limited or those included were not relevant to crisis situations. Although the absence of a suitable preference-based quality of life measure means that quality adjusted life years (QALYs) cannot be included in the analyses, recent reports describe how wellbeing can be valued and compared to QALYs. Novel approaches emerging from that research will be incorporated into our analyses. Economic analyses will focus on secondary care health service use. Administrative- and individual-level data will be linked to provincial cost data to establish costs for participants who received care in the pre-implementation and maintenance phases. Although our care bundle is likely to have an impact on primary care health services and other areas of public expenditure, the scope of this project does not permit us to measure the impact of the bundle on areas outside of health; assigning costs to those outcomes would be problematic.

Individuals at the Institute of Health Economics will perform an economic analysis of the intervention by comparing the healthcare resource use and costs during the pre-bundle implementation period and post-bundle implementation period. The economic analysis will use administrative data **(hospitalizations, outpatient visits, and physician claims, between January 2020 and June 2022; and population registry data between April 1, 2019 and March 31, 2023).**

4. COMMUNICATION PLAN

The communications plan, outlining our efforts and recruit participants for this project, will be led by the project lead, co-lead, and operational leads for clinical care settings. The project manager will operationalize the plan, as it will be linked to the project timeline and change management plan. The communications objectives are outlined below by study year.

4.1 Year 1

1. Increase understanding among ED and clinic healthcare providers of the project's purpose, how the bundle will be introduced, its impact on workflow/roles/responsibilities, and its benefits
2. Establish lines of communication with partners (primary healthcare, school- and community-based services) and families on how the bundle enhances healthcare availability/access, will be evaluated, offers families opportunities to participate in evaluation, and enables inter-agency collaborations
3. Develop communication strategies for partners and families by consulting on stakeholder communication preferences

To facilitate meeting these key objectives, operational leads will hold quarterly meetings with healthcare providers to discuss and optimize the bundle. Practical training sessions on bundle components (e.g., ASQ and HEADS-ED, CAPA) will be held between meetings, which allow us to collect input from healthcare providers. In partnership with stakeholder team members, operational leads, and patient partners we will host webinars and develop project communication materials. We will create and test plain language materials for families.

4.2 Year 2

Year 2 will focus on communication strategies for bundle implementation:

1. Among healthcare providers: introduce change management process maps, discuss resistance to change, and review key performance indicators from PDSA cycles
2. Among partners: refine strategies for communication and collaboration based on Year 1 experience and feedback
3. Among families: clarify bundle misconceptions/misunderstandings by refining community engagement campaign (e.g., online media, posters, print materials)
4. Among team members and collaborators: maintain regular communication between on project progress, milestones and input

The focus will be on implementing change management plans and PDSA cycles, and obtaining feedback from children, youth, and parents during PDSA cycles on the bundle, as well as holding regular team meetings and obtaining feedback from partners on the strength of partnership and communication. In the bundle maintenance phase (part of Year 2 and Year 3), key to communication will be sharing results and recommendations in real-time to share progress being achieved.

4.3 Year 3

1. Create products tailored to stakeholders that communicate results and recommendations
2. Share finalized change management documents
3. Hold meetings to discuss potential province-wide bundle deployment (e.g., eligible EDs, AMH SCN, ESCN)

5. PATIENT ENGAGEMENT

Patient engagement is central to this project. We are using Amirav's adaptation of the International Association of Public Participation Spectrum of Engagement for health research and the CIHR-SPOR Patient Engagement Framework as the basis for our evidence-informed approach to engagement. These resources and our consultation with Jananee Rasiah (Patient Engagement Platform, Alberta SPOR SUPPORT Unit) shaped an engagement framework that guides team activities.

Youth and parents/caregivers are rarely engaged meaningfully across the research continuum. In contrast, as core members of our team, patient partners will help oversee bundle introduction to EDs, interim data analyses, and Plan-Do-Study-Act (PDSA) cycles, all of which will have a strong focus on learning from family experiences during initial implementation. Patient partners will also be involved in governance activities (see Section 7.0 for further details) and key knowledge translation (KT) activities—to incorporate their perspectives, they will help write project reports and updates that will be distributed to partners and stakeholders.

6. CHANGE MANAGEMENT

In consultation with AHS Change Adoption & Evaluation, we will apply the ADKAR® model to prepare for change. The Prosci ADKAR® Model is a goal-oriented change management model that guides individual and organizational change. ADKAR is an acronym that represents the five tangible and concrete outcomes that our team will follow to achieve lasting change: *awareness, desire, knowledge, ability, reinforcement.* By outlining the goals and outcomes of successful change, the ADKAR Model will equip our team's leaders to facilitate institutional change, and it will support our team members and the effected healthcare providers at all study sites throughout the change. PRIHS funding will support Change Management Leads in Calgary and Edmonton with the knowledge and abilities to implement our care bundle. Change management teams incorporate a Lead, Change Specialist, project team members and champions—ED and clinic managers, patient care managers, nurse educators, and senior leaders.

Below, we use of Normalization Process Theory (NPT) as a framework to guide change and 'normalize' the bundle into routine acute mental health and addictions care of children and youth. NPT is used to support implementation of complex clinical interventions and new care collaboratives—our project features both these aspects. It includes four constructs for the work that is required to fully implement a set of new practices: coherence, cognitive participation, collective action, and reflexive monitoring. Each construct is a distinct phase of implementation. Our approach will be non-linear—while we will begin with coherence and continue through the phases sequentially, we will revisit constructs to address project needs and challenges that occur over time.

6.1 Phase 1: Coherence (Year 1)

The milestone for Phase 1 includes establishing awareness of the proposed bundle of care. Change Management Leads will support and coach all management levels, overseeing integration of change management activities. Change Specialists (reporting to Leads) will complete all change management deliverables. Specialists will work with champions on training plans and change management strategies, identifying how the care bundle will impact individuals, what the bundle expects from each stakeholder group, how to support each group for change, and roles of champions. The strategy will be developed in Year 1, informed by champions' identification of risks, barriers, and enablers to introducing and supporting the care bundle in AHS, and supported by a change management communication plan. Each Change Specialist and their change team will create change management process maps, champion roadmaps, coaching and training plans for frontline providers along with a resistance management plan to facilitate awareness, desire, and knowledge.

We will establish awareness among healthcare providers, managers, directors and administrative staff in:

- Alberta and Stollery Children's Hospital EDs
- Existing AHS children's mental health clinics (1.5 in Calgary; 4 + 2 x 0.5 in Edmonton and surrounding area [6 clinics with 0.5 to 1.0 availability])

- Mental health and addictions services offered in primary care, schools, community-based and non-profit organizations

Change management plan

Our change management approach will define awareness, desire and knowledge (as per ADKAR®) among individuals directly (e.g. ED physicians and nurses, urgent clinic therapists) and potentially affected (e.g. primary healthcare providers) by the bundle. With support from project managers in Calgary and Edmonton, operational leads will hold information meetings (webinars, teleconferences, in-person meetings) with these individuals. Leads will use the NPT Tool Kit to discuss how the bundle will be introduced to each setting and its impact on workflow and roles/responsibilities. The Tool Kit is interactive and accessible online, providing collaborators and stakeholders with a structured process to consider a range of issues related to implementation processes—issues may vary depending on how and to what extent different individuals expect to interact with the bundle, and how current practices differ from the proposed bundle.

Communication plan

Key communication objectives are to: (1) increase understanding among ED and clinic healthcare providers of the project's purpose, how the bundle will be introduced, its impact on workflow/roles/responsibilities and its benefits; (2) establish lines of communication with partners (primary healthcare, school- and community-based services) and families on how the bundle enhances healthcare availability/access, will be evaluated, offers families opportunities to participate in evaluation, and enables inter-agency collaborations; and (3) develop communication strategies for partners and families by consulting on stakeholder communication preferences.

6.2 Phases 2 and 3: Cognitive participation and collective action (Year 2)

Cognitive participation refers to how and to what extent individuals engage themselves and others in implementing the bundle of care. Fully implementing and normalizing our bundle requires key changes to clinical practice through collective action across multiple healthcare providers and partners. Phases 2 and 3 will occur concurrently when we begin bundle implementation (Year 2; month 3) and will involve the individuals in settings targeted in Phase 1 as well as individuals providing care in the 2 new urgent care clinics. The key milestone for these phases will be relational work to build and sustain a community of practice with two key stakeholder groups:

Among key individuals in EDs, new acute care clinics, and AHS mental healthcare clinics

During PDSA cycles, operational leads will consult with frontline healthcare providers, unit/patient care managers, and project champions to identify how healthcare practices and processes are influenced over time. Resistance or lack of engagement will also be explored and addressed using change management process maps, champion roadmaps, coaching and training plans for frontline providers, and a resistance management plan (see Change Management Approach). Operational leads will hold teleconferences to share findings across care settings, and collectively determine how best to support changes while mitigating barriers and challenges to change. This activity is noteworthy as it reflects the first coordinated effort to align efforts in Calgary and Edmonton for child and youth acute mental health and addictions care.

Among key individuals working in mental health and addictions services offered in primary care, schools, community-based and non-profit organizations

Operational leads will continue to hold quarterly meetings with these individuals; agendas for meetings will be co-created (leads + stakeholders) and informed by findings from the PDSA cycles. In general, meetings will focus on ensuring choice and partnership for families and maximizing access and availability of services in the post-crisis period for families. Meetings will conclude with action items to revise protocols, processes, or resources to ensure these foci are fully operationalized.

The tools completed in Year 1 (e.g., change management process maps, champion roadmap, coaching and training plans for frontline providers, resistance management plan) will support bundle implementation in Years 2 and 3. Change Specialists will create an electronic database to track and report adoption issues and progress of change. Completed deliverables will be integrated into the project plan before implementation and reviewed quarterly for responsiveness to individual and organizational needs. Data from PDSA cycles and project-specific performance indicators will help guide review.

6.3 Phase 4: Reflexive monitoring (Year 3)

We will assess our implementation processes in the EDs and clinics using PDSA cycles, our supporting change management strategy, and our communication plan to determine their effectiveness and usefulness with frontline healthcare providers, unit/patient care managers, directors, and administrative staff. To gauge the strength and stability in our partnerships with key individuals involved in primary healthcare, schools, community-based and non-profit organizations, we will ask them to complete the VicHealth Partnerships Analysis Tool at project onset and every 6 months to highlight relationship strengths and identify areas for improvement.

Change Specialists and champions will identify project-specific performance indicators (organizational, such as adherences to project plan; individual, such as ADKAR® Model surveys). Indicators will measure behaviour changes and monitor effectiveness of the change management approach. Change management activities will be tracked. At project end, our change management strategy will be reviewed and refined. Change Specialists and champions from each setting/stakeholder group will ensure that change management under their leadership moves the bundle of care from a funded research project to a component of AHS operations by Year 3. The final change management strategy will be shared with others considering bundle implementation.

7. ETHICAL CONSIDERATIONS

The study will be presented to the University of Calgary and University of Alberta Research Ethics Boards. Data collection will only commence after approval is obtained. Contact information will be collected at ED registration at each study site for eligible study participants. All children/youth eligible for the study will be given a brief study information sheet at triage, which will include an opt-out mechanism for those who prefer not to be contacted. Research assistants will conduct daily reviews of ED medical records to identify eligible patients. Eligible patients and their parents/caregivers who do not opt-out of being contacted will be contacted by the research team by telephone to obtain informed consent (or assent as appropriate). Individuals will only be enrolled after free and informed consent/assent has been received. Parents/caregivers will be provided with the contact information for the responsible investigator for the given site (Newton: Stollery Children's Hospital; Freedman: Alberta Children's Hospital). During the project, all information provided by participants will be held in confidence and no identifying

information will be published. Participants will be free to withdraw at any time and will receive the same standard of mental healthcare.
