# Supplementary material for: Emergency Department Pediatric Mental Health Care Bundle and Family Quality of Life
Source: JAMA Netw Open. 2025 Dec 9;8(12):e2548860. doi: 10.1001/jamanetworkopen.2025.48860 (PMC12690423; doi:10.1001/jamanetworkopen.2025.48860)
Supplement: Supplement 3. — Nonauthor Collaborators [file jamanetwopen-e2548860-s003.pdf]

Supplemental Online Content: Nonauthor Collaborators
*First name, last name, and suffix (if applicable) are required and will appear in PubMed.

*Group Name(s): Pediatric Emergency Research Canada (PERC)							
***First Name and Middle Initial(s)**	***Last Name**	***Suffix (eg, Jr, III)**	Academic Degrees	Institution	Location (city, state/province, country)	Role or Contribution, eg, chair, principal investigator	Group (if more than 1 Group listed in the byline) and/or Subgroup (eg, Steering Committee)
Samina	Ali		MDCM	University of Alberta	Edmonton, Alberta, Canada	Chair	
Jocelyn	Gravel		MD, MSc	Le Centre hospitalier universitaire Sainte-Justine	Montreal, Quebec, Canada	Executive Board Member	
Vikram	Sabhaney		MD	BC Children's Hospital	Vancouver, British Columbia, Canada	Executive Board Member	
Tyrus	Crawford		BSocSc	Children's Hospital of Eastern Ontario	Ottawa, Ontario, Canada	Research Coordinator Representative	
Andrea	Eaton		MSc, BScN	University of Alberta	Edmonton, Alberta, Canada	National Cooredinator	
Gabrielle	Freire		MSc, MD	University of Toronto	Toronto, Ontario, Canada	Executive Board Member	
Henry	Li		MD	University of Alberta	Edmonton, Alberta, Canada	Fellow Representative	
Naveen	Poonai		MSc, MD	Western University	London, Ontario, Canada	Vice Chair	
Bruce	Wright		MD	University of Alberta	Edmonton, Alberta, Canada	Executive Board Member	
Roger	Zemek		MD	University of Ottawa	Ottawa, Ontario, Canada	Past Chair	
