# Supplementary material for: Emergency Department Pediatric Mental Health Care Bundle and Family Quality of Life
Source: JAMA Netw Open. 2025 Dec 9;8(12):e2548860. doi: 10.1001/jamanetworkopen.2025.48860 (PMC12690423; doi:10.1001/jamanetworkopen.2025.48860)
Supplement: Supplement 4. — Data Sharing Statement [file jamanetwopen-e2548860-s004.pdf]

Data Sharing Statement

Newton. Emergency Department Pediatric Mental Health Care Bundle and Family Quality of Life. *JAMA Netw Open*. Published December 09, 2025.
doi:10.1001/jamanetworkopen.2025.48860

Data
Additional Information: Clinicaltrials.gov https://clinicaltrials.gov/study/NCT04292379?intr=NCT04292379&rank=1 NCT04292379
Data available: No

Additional Information
Explanation for why data not available: The study was not approved to provide individual patient data outside of study team members.
